# Death of Marcus L. Taft, M. D.

**Published:** 1850-04

**Authors:** 


DIED.

Marcus L. Taft, M. D., at the Quarantine Hospital, Long Island, of typhus fever, in his 29th year. Those of our readers who were present at the last meeting of the American Medical Association in Boston, will remember this young gentleman as the reader of the Report on Medical Education, and will regret with us, that a career that promised so brightly, should have been so prematurely brought to a close.

